# The Treatment of Sleeplessness Complicating Other Diseases

**Published:** 1892-08-20

**Authors:** 


### THE PRACTITIONER'S MIRROR AND RETROSPECT.

[*The Editor will be glad to receive offers of co-operation and contributions from members of the profession. All letters should be addressed to* The Editor, The Lodge, Porchester Square, London, W.]

### MEDICINE.

### THE TREATMENT OF SLEEPLESSNESS COMPLICATING OTHER DISEASES.

It is not intended in this article to deal with insomnia as a complaint by itself, but rather briefly to discuss its treatment in the case of patients who are already suffering from other disorders, and for whom it is of importance to procure a good night's rest.

In many instances—as, for example, in the course of a bad case of pneumonia—a good sleep will do more than anything else to improve the patient's condition; and it is scarcely too much to say that on the recognition or neglect of this fact, and on the success or failure of the treatment adopted, will not infrequently depend the issue between life and death in cases of acute illness.

In all severe cases the successful treatment of sleeplessness will depend both on the physician and on the nurse; and it is certain that, without the aid of a skilled nurse, the measures adopted by the physician will often be of no avail. There are numerous plans and devices, varying with the nature of the case and the condition of the patient, which will suggest themselves to a good nurse, and which may even obviate the necessity for medical interference. With these, however, we cannot now deal.

The following list includes most of the hypnotics in ordinary use; and to each we add a short account of its more prominent characteristics:—

1. Potassium Bromide.—There is probably no drug which is more commonly prescribed for insomnia than bromide of potassium, and yet it is one of those which, we think, gives the least satisfactory results. Its properties are so well known that we need scarcely say more than that it is only the milder cases of sleeplessness that will yield to its influence; nevertheless, it acts as a sedative, and is often of value as such, especially when given in small and repeated doses. It is frequently combined with chloral hydrate, but we have never been able to satisfy ourselves that it materially increases the efficacy of that drug. The depressing mental effect produced in many people by its use must be borne in mind; indeed, in some cases of nervous depression, its administration has to be abandoned on that ground.

2. Chloral Hydrate.—In a certain class of cases there is no drug so useful and so reliable as chloral hydrate. Its depressing action on the heart forbids its use where there is much disease of that organ, whilst the unpleasant effects often noticed the following day, namely, headache, mental depression, &c., are points against its employment where other hypnotics can be substituted. It is in cases of delirium with excitement—as, for instance, in delirium tremens—that the use of this drug is especially indicated; and in these we know of none other which can be used with advantage in its place. For this purpose, however, it must be prescribed in large and repeated doses, until sleep is procured, a course which may safely be followed if the chloral be combined with correspondingly large doses of tincture of digitalis. It is needless to say, however, that a careful watch must be kept on the pulse during its administration. It may be added that in all cases the addition of a little digitalis will enable larger doses of the drug to be given with safety than could otherwise be employed, and will tend to counteract its unpleasantly depressing effect. As already stated, we do not consider that there is any appreciable advantage to be gained by the addition of bromide of potassium.

Coming now to the hypnotics more recently introduced, there are four which may be mentioned as being of considerable value:—

3. Sulphonal.—This drug is a useful hypnotic in ordinary cases of insomnia. As a rule it produces no bad effects, though it is said occasionally to cause slight cardiac depression, and its use may be temporarily followed by the appearance of a rash. We have, however, never experienced any such result. It may be given in doses of 15, 20, or 30 grains; it is very slightly soluble in water, and must therefore be made up with mucilage. If given in cold water, its action is often much delayed, and it is well to administer it at least an hour before its effect is desired; indeed, at times, it fails to produce sleep until several hours after it has been taken. A better plan is to give it in hot water, whereby its solubility, and therefore its rapidity of absorption, are considerably increased.

4. Chloralamide acts more quickly than sulphonal, and, we think, more certainly. It is supposed to possess all the advantages, and none of the disadvantages, of chloral hydrate. The latter part of this statement is certainly true, and chloralamide may be given with perfect safety, and without fear of producing any unpleasant after-effects; it acts, however, as a very poor substitute for chloral in the cases of delirium mentioned above, and is decidedly the weaker drug of the two. It may be given in rather larger quantities than sulphonal, ʒss. being an ordinary dose for an adult. It is only slightly soluble in water, but is readily soluble in spirit (1 in 2); it is often given in milk, but is better dissolved in a little brandy. At a moderate heat (120° F.) it is decomposed, and it must not therefore be given in hot water. Like chloral, it occasionally produces a rash.

5. Of Urethane we have but little experience. It is said to be a safe hypnotic, and to act sometimes when other drugs have failed; but this statement may be safely made of any other drug. We may mention, however, that we have seen one case (peripheral neuritis) in which urethane was the only medicine which produced any beneficial effect. It may be given in doses of 10 to 20 grains, or more, and is readily soluble in water.

6. Paraldehyde is, in our opinion, more reliable than any of the three last-mentioned drugs, and its use is perfectly free from risk. It acts speedily, as a rule—often within half an hour of its administration, and causes no unpleasant effects the following day. Half a drachm is an ordinary dose, but this may safely be doubled in the more obstinate cases of insomnia. Alone of the four drugs just described, it has some effect in cases of delirium; but, for this purpose, ʒi. is the smallest dose that should be ordered; indeed, we are told that in some asylums as much as ʒii. is not infrequently given at once. Paraldehyde is a somewhat oily liquid, with a strong and rather nauseous odour and disagreeable taste. Given in water (ʒi. to ℥i.), it requires to be well shaken up for some time before a proper mixture can be obtained. It is better, however, to give it in a little brandy or wine, with which it mixes better, and by which its unpleasant taste is to some extent hidden. It is considerably cheaper than any of the three preceding drugs.

7. Opium.—None of the hypnotics hitherto described are to be relied on where much pain is present. Sulphonal, chloralamide, urethane, and paraldehyde are especially powerless in this respect, and we are driven in most cases, where local anodyne treatment fails or is impracticable, to fall back upon the use of opium or morphia. With its anodyne action we are not now concerned, but we would point out that, as a simple hypnotic, opium, under certain circumstances, is of immense value. In cases of acute illness, with much restlessness and nervous disturbance, its sedative effect, in addition to its hypnotic action, is most beneficial. Often it is most advantageous to give a very small dose, and repeat it, if necessary, in an hour; in other more severe cases a single larger dose will act best. The few cases in which small doses of opium are dangerous will easily be recognised beforehand, and for these, of course, the above treatment is unsuitable.

8. Alcohol.—Sometimes no hypnotic succeeds so well as alcohol; ℥ss. of brandy, in hot water, taken last thing at night, will occasionally send the patient to sleep when all other drugs have failed to do so. We have most often seen this result in cases of advanced heart disease, where insomnia is a prominent and very distressing feature; cases of chronic Bright's disease are also not uncommonly benefited by the same treatment. Even should it fail to cause sleep, a little brandy may often enable the patient to pass a more com. fortable night than he would otherwise have done; but we need scarcely say that its effect varies inversely with the amount taken regularly during the day.

In conclusion, it must be remembered that it is often impossible to say beforehand which of these various hypnotics will act in any individual case, and that it may be necessary to change the drug as its effect wears off with repetition.

